# GENDER DIFFERENCES IN THE PATHWAYS FROM DAILY STRESS TO CARDIOVASCULAR HEALTH IN CAREGIVERS

**DOI:** 10.1093/geroni/igae098.4198

**Published:** 2024-12-31

**Authors:** Kelsey Biernot, Erin Clancy, Rachel Koffer

**Affiliations:** Arizona State University, Tempe, Arizona, United States; Arizona State University, Tempe, Arizona, United States; Arizona State University, Phoenix, Arizona, United States

## Abstract

Caregivers’ risk for cardiovascular disease may be increased because of stress. Furthermore, less is known about gender differences in the effects of caregiving on stress and cardiovascular health. This study examined the associations between gender and daily stressors on cardiovascular health in caregivers, first cross-sectionally at two time points 10 years apart and then longitudinally. Data from two waves of the Midlife in the United States Study (Wave 2: n = 2,087, 13.56% caregivers; age = 33-84 years; Wave 3: n= 3,437, 13.73% caregivers; age= 39-93 years) included caregiving status in the past 12 months, daily diary stressor reports, measures for the American Heart Association’s Life’s Simple 7. Cross-sectional regressions found caregiving was only related to increased stressors in women, not in men, at Wave 2 (BWave2 = -0.08, p= 0.02; BWave3 = -0.01, p= 0.84). Both waves showed significant interactions between stressor exposure and caregiving status on cardiovascular health (BWave2 = 0.46, p = 0.047; BWave3 = -0.95, p< 0.001). In Wave 2, caregiving was related to lower cardiovascular health for those with more stressors, while in Wave 3 caregiving was related to worse cardiovascular health for those with fewer stressors. The interactive effects of caregiving and stressor exposure did not differ by gender. Follow-up analyses revealed the effects of wave 2 caregiving and stress did not relate to wave 3 cardiovascular health. Findings indicate caregiving status is most important for concurrent cardiovascular health, and differs by the context of daily stressor experiences.

